# Letrozole increases preantral follicle growth and decreases estradiol production without impairing follicle survival

**DOI:** 10.1186/s13048-022-01073-2

**Published:** 2022-12-24

**Authors:** Fukiko Kasuga-Yamashita, Tsuyoshi Baba, Sachiko Nagao, Yuya Fujibe, Miyuki Morishita, Yoshika Kuno, Tasuku Mariya, Hiroyuki Honnma, Toshiaki Endo, Tamotsu Kiya, Tsuyoshi Saito

**Affiliations:** 1 Department of Obstetrics and Gynecology, Sapporo Medical University, South 1 West 16, 060-8543 Sapporo, Hokkaido Japan; 2 Sapporo ART Clinic, 1-2 North 7 West 4, 060-0807 Sapporo, Hokkaido Japan; 3 Ena Asabu ART Clinic, 2-2-7 Asabu, 001-0045 Sapporo, Hokkaido Japan

**Keywords:** letrozole, polycystic ovary syndrome (PCOS), follicular development, growth factors, ovarian hyperstimulation syndrome (OHSS)

## Abstract

**Background:**

Letrozole has been reported to be effective in treating anovulation, preventing ovarian hyperstimulation syndrome (OHSS), and retrieving oocytes in breast cancer patients. However, the role and mechanism of letrozole in follicular development remain unclear.

**Results:**

We treated mouse preantral follicles with various treatments; we found no significant difference in follicle survival rates in the letrozole (LET) group compared with the control group, but the average diameter of follicles in the LET group tended to be larger (CTRL vs. LET 30, *p* = 0.064; CTRL vs. LET 100, *p* = 0.025). The estradiol concentrations in culture media of the LET group were significantly lower than those observed in the control group (CTRL vs. LET 30, *p* = 0.038; CTRL vs. LET 100, *p* = 0.025). We further found a marked increase in follicle-stimulating hormone receptor (FSHR) gene expression in response to letrozole treatment (CTRL vs. LET 30, *p* = 0.075; CTRL vs. LET 100, *p* = 0.034). This result suggested that increased FSHR expression promotes follicle development. Letrozole inhibited aromatase activity, but the effect was limited. Letrozole did not significantly reduce vascular endothelial growth factor (VEGF) gene expression.

**Conclusions:**

Letrozole may promote follicle development by increasing the expression of FSHR. Letrozole may be useful for fertility preservation of patients with estrogen-dependent cancers such as breast cancer and various other cancers. Whether letrozole has a direct effect in reducing OHSS requires further investigation.

## Background

Polycystic ovary syndrome (PCOS) is the most common endocrine disorder in women of reproductive age and often leads to infertility. Several criteria have been suggested for diagnosing PCOS, and the prevalence of PCOS varies from 8.7–17.8% in accordance with the different diagnostic criteria [1, 2]. PCOS is characterized by hyperandrogenism, ovulation disorder, and polycystic ovarian morphology. While the etiology of PCOS is unknown, excess androgens are thought to be a critical factor driving the pathology of PCOS [3–5].

Various management strategies have been proposed for infertile women with PCOS, but no definitive treatment has been established [6, 7]. Controlled ovarian stimulation (COS) with gonadotropins is used in assisted reproductive technology (ART) and leads to a higher risk of ovarian hyperstimulation syndrome (OHSS) [8]. The clinical manifestation of OHSS includes abdominal tenderness and swelling, which are caused by an increased vascular permeability and effusion to the extravascular space. Vascular endothelial growth factor (VEGF) plays a crucial role in the development of OHSS [9, 10]. At present, no effective methods for OHSS treatment have been established, and therefore prevention is critical.

Fertility preservation for patients with various cancers has become an important issue in recent years [11, 12]. Many cancer patients of childbearing age develop ovarian failure in response to radiotherapy and chemotherapy [13], and the patients are often forced to give up their pregnancies. Many patients need embryo or oocyte cryopreservation before cancer treatment. Therefore, establishing a safe and effective method of ovarian stimulation is critical. Breast cancer is the most commonly diagnosed malignancy in women of childbearing age. A major concern regarding fertility preservation is the exposure of patients to high amounts of estrogen during COS, since approximately two-thirds of breast cancer patients have estrogen-receptor-positive cancer, and estrogen promotes the growth of breast cancer cells [14].

In recent years, letrozole has attracted attention as an infertility treatment [15–18]. Letrozole is a targeted aromatase inhibitor that has been primarily used in post-menopausal women with breast cancer. It inhibits estrogen production by ovaries and subsequently interrupts the negative feedback action of estrogen on follicle stimulating hormone (FSH) production [19]. In this manner, letrozole increases FSH and indirectly promotes follicle development. However, estrogen depletion is also detrimental to follicle development [20]. Data on the effects of letrozole on follicle dynamics are limited. Additionally, some studies have reported that letrozole may suppress VEGF production and it can reduce the incidence of OHSS [21, 22]. However, whether letrozole affects follicles and influences or decreases the incidence or severity of OHSS has not been elucidated. In this study, we examined the direct effects of letrozole on murine secondary follicle development and the effect on the prevention of OHSS.

## Results

**Fig. 1:**
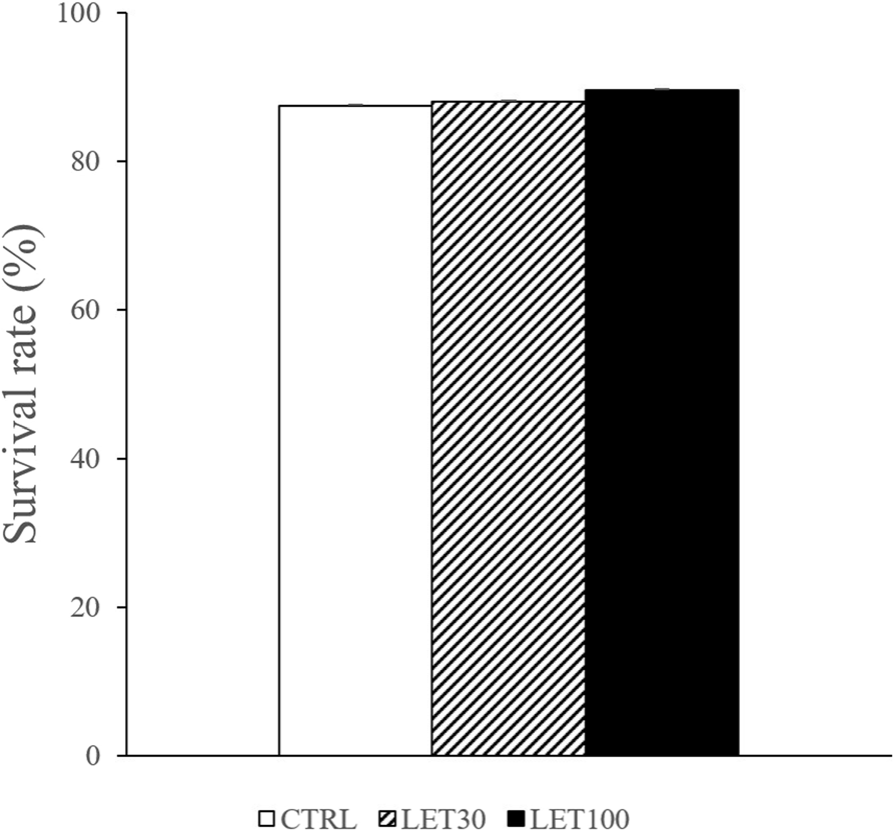
Follicle survival rates in the control (CTRL), letrozole (LET) 30 ng/ml, and LET 100 ng/ml treatment groups at day 10 of culture. CTRL group, base media plus letrozole vehicle (dimethyl sulfoxide, DMSO); LET 30 group, base media plus 30 ng/mL letrozole and DMSO; and LET 100 group, base media plus 100 ng/mL letrozole and DMSO. Data are expressed as mean ± standard errors. There was no significant difference in follicle survival rates among the groups (*P* = 0.247, one-way ANOVA)

**Fig. 2:**
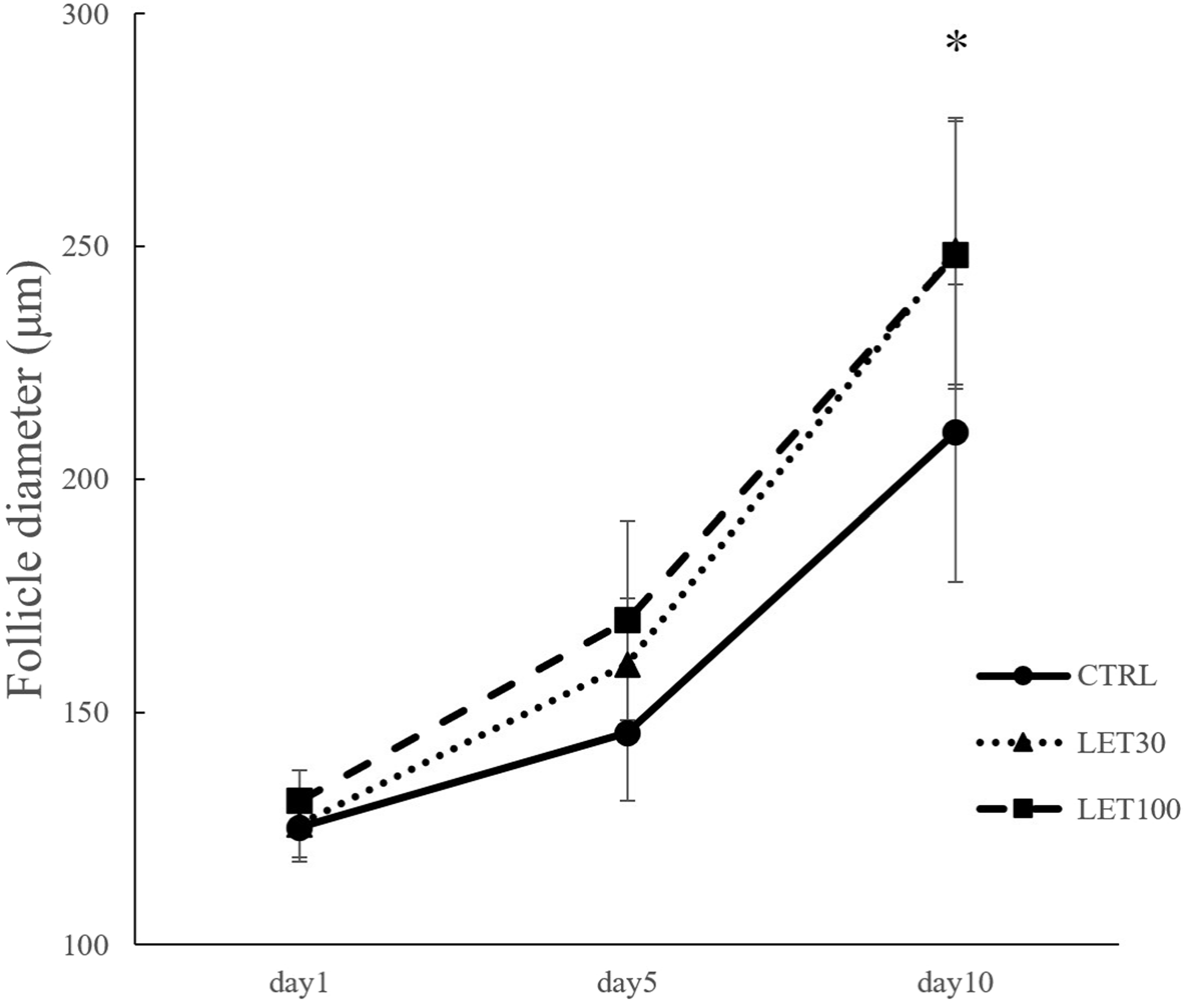
Effect of letrozole (LET) on average follicle diameters. Follicle growth was monitored at the indicated time points. Follicles were treated as described in Fig. 1. Data are expressed as mean ± standard errors. *P < 0.05, one-way ANOVA and Student–Newman–Keuls post hoc analysis

We examined the effects of letrozole on murine secondary follicle development. We evaluated follicle survival rates and diameters in the control (CTRL), letrozole (LET) 30 ng/ml, and LET 100 ng/ml treatment groups at day 10. Follicle survival rates of all groups were between 87% and 89%; there was no significant difference among the groups (*P* = 0.247) (Fig. 1). The average diameter of follicles in the LET 100 ng/ml group was significantly larger than that observed in the CTRL group (248.11 ± 57.43 µm vs. 209.95 ± 65.11 µm, respectively; *P* = 0.025). The follicle diameter in the LET 30 ng/ml group (249.04 ± 58.57 µm) tended to be larger than that observed in the CTRL group (*P* = 0.064) (Fig. 2). The rates of follicles above 250 µm in size, which might be a threshold of antrum formation [23], was 39.3% in CTRL, 43.5% in LET 30 ng/ml, and 60.0% in LET 100 ng/ml.

**Fig. 3:**
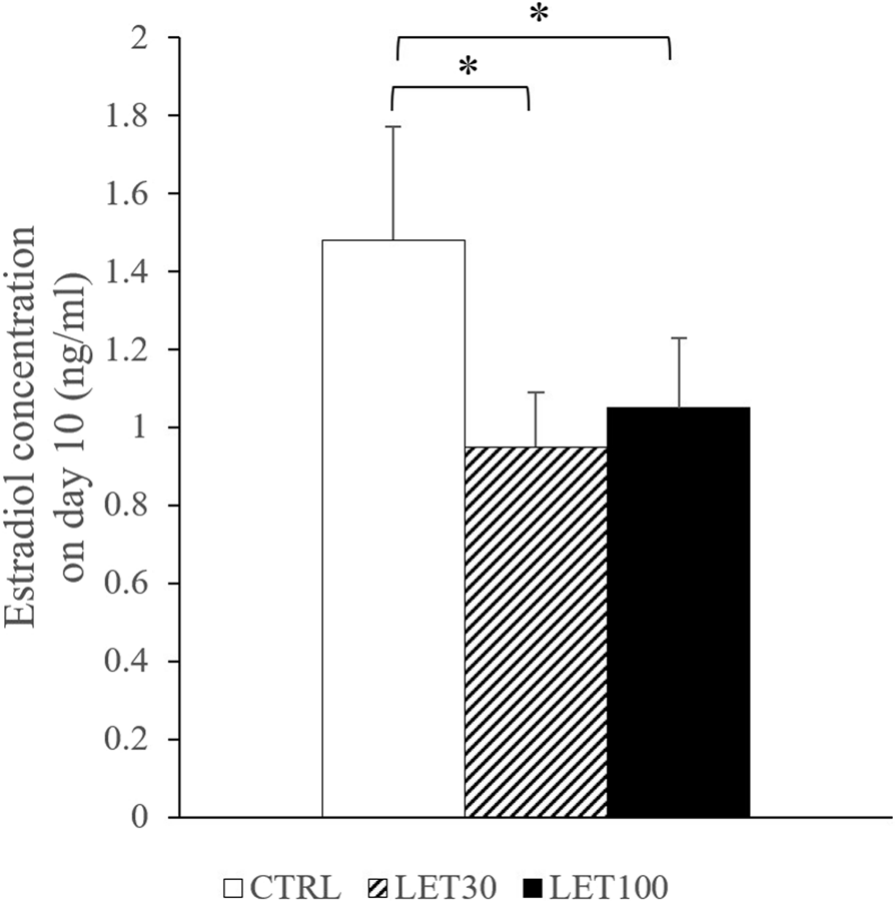
Estradiol (E2) concentrations in culture media at day 10. Follicles were treated as described in Fig. 1. Data are expressed mean ± standard errors. *P < 0.05, by one-way ANOVA and Student–Newman–Keuls post-hoc analysis

At day 10, estradiol (E2) concentrations in the culture media were 1.48 ± 0.57 ng/mL in the CTRL group, 0.95 ± 0.27 ng/mL in the LET 30 ng/ml group, and 1.05 ± 0.37 ng/mL in the LET 100 ng/ml group (Fig. 3). The E2 concentrations in culture media of the LET 30 ng/ml and LET 100 ng/ml group were significantly lower than those observed in the CTRL group (*P* = 0.038 and *P* = 0.025, respectively). No concentration-dependent difference in E2 concentration was observed.

**Fig. 4:**
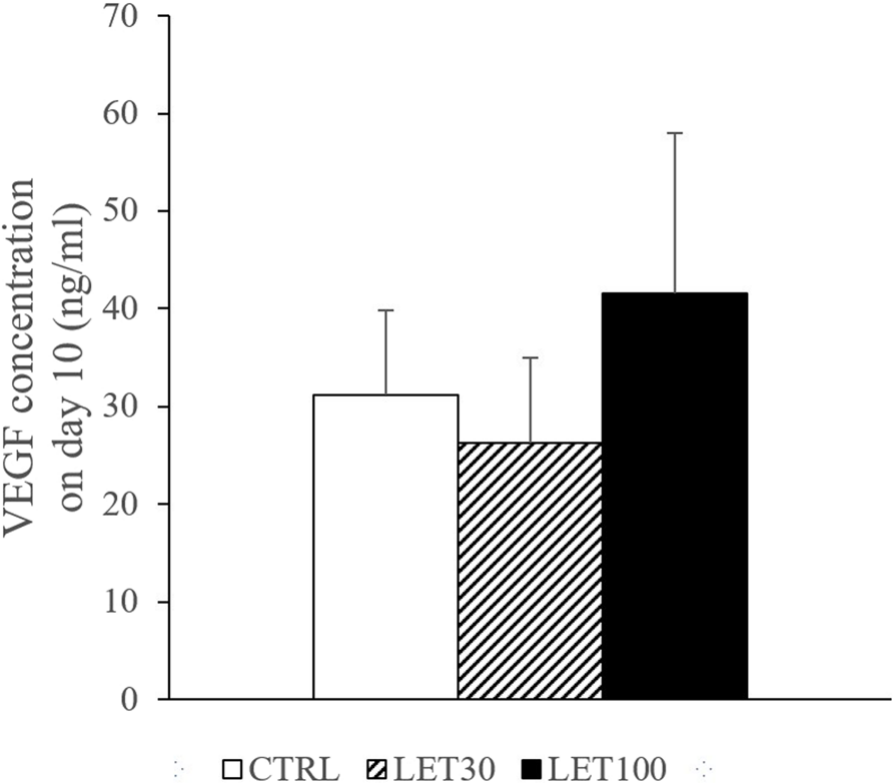
Vascular endothelial growth factor (VEGF) concentrations in culture media at day 10. CTRL group, base media plus letrozole vehicle (dimethyl sulfoxide, DMSO); LET 30 group, CTRL media plus 30 ng/mL letrozole and DMSO; and LET 100 group, CTRL media plus 100 ng/mL letrozole and DMSO. Data are expressed mean ± standard errors. There was no significant difference in VEGF production among these groups (*P* = 0.374, one-way ANOVA)

VEGF concentrations in culture media on day 10 were 31.09 ± 17.48 pg/mL in the CTRL group, 26.31 ± 17.28 pg/mL in the LET 30 ng/ml group, and 41.54 ± 32.83 pg/mL in the LET 100 ng/ml group (Fig. 4). There was no statistically significant difference among the three groups (*P* = 0.374).

**Fig. 5:**
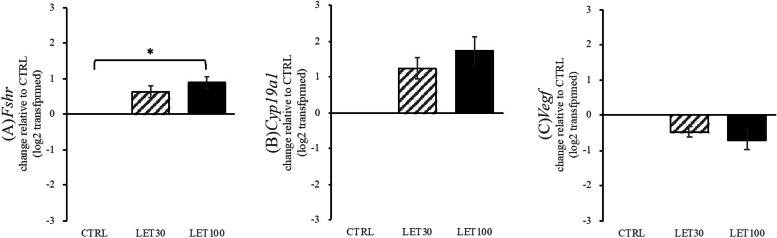
Effects of letrozole (LET) on gene expression of hormone receptors, *Fshr*, *Cyp19a1*, and *Vegf*. Isolated follicles were cultured for 10 days and then analyzed for gene expression of (**A**) *Fshr*, (**B**) *Cyp19a1*, and (**C**) *Vegf*. CTRL group, base media plus letrozole vehicle (dimethyl sulfoxide, DMSO); LET 30 group, CTRL media plus 30 ng/mL letrozole and DMSO; and LET 100 group, CTRL media plus 100 ng/mL letrozole and DMSO. Data are expressed as mean ± standard errors and have been log_2_ transformed. LET 30 and LET100 groups were compared with the control. *P < 0.05, by one-way ANOVA and Student–Newman–Keuls post-hoc analysis

The relative mRNA levels of FSH receptor (FSHR), aromatase (*Cyp19a1*), and VEGF in the experimental groups are shown in Fig. 5. *Fshr* mRNA was significantly higher in the LET 100 ng/ml group than that observed in the CTRL group (*P* = 0.034). *Fshr* mRNA levels in the LET 30 ng/ml group tended to be higher than that observed in the CTRL group, but there was no significant difference (*P* = 0.075). C*yp19*, subfamily a, polypeptide 1 (C*yp19a1*) mRNA levels in the LET groups tended to be higher than that in the CTRL group, with no significant difference (*P* = 0.072) (Fig. 5). *Vegf* mRNA in the LET groups tended to be higher than that observed in the CTRL group, but there was no statistically significant difference among these groups (*P* = 0.089) (Fig. 5).

## Discussion

In this study, we found that letrozole promoted the growth of secondary follicles without affecting survival. Letrozole also suppresses E2 production without a negative effect on follicular development. This result suggests that letrozole may be a potential candidate for follicular stimulation for infertility treatment and fertility preservation. Our results showed that letrozole significantly decreased E2 production; however, the decrease was not as drastic as expected. We speculate that E2 levels are initially decreased by letrozole but eventually return close to normal levels as a long-term consequence of the increased *Cyp19* gene expression and testosterone synthesis (the primary substrate for E2) [24]. It may be theoretically reasonable to use combined letrozole and tamoxifen in egg retrieval in breast cancer patients because tamoxifen inhibits E2 to bind to E2 receptor. However, tamoxifen also increases E2, and thus studies are needed to elucidate the effects of combined use of letrozole and tamoxifen on follicle dynamics.

To explore the potential molecular mechanism of letrozole on follicle development, we evaluated the expressions of various genes related to follicle development. Our results revealed a significant increase in FSHR gene transcription in response to letrozole administration. This result suggested that increased FSHR expression potentiates FSH actions and subsequently promotes follicle development. This finding is in accordance with previous studies showing that letrozole not only exerts a negative feedback effect on the pituitary gland (increased secretion of FSH) from estrogen depletion, but it also induces the accumulation of testosterone in the follicles and subsequent increase in FSHR expression [25, 26] Several studies reported decreased oocyte maturation rates in COS with letrozole [27, 28]. Therefore, the follicle diameter in response to human chorionic gonadotropin (hCG) trigger should be larger than that in standard COS to obtain equivalent mature oocytes [29]. It may delay the hCG trigger and oocyte retrieval, which is not acceptable in patients with cancer. Accelerated follicle growth by letrozole may counterbalance the delay of hCG trigger.

Letrozole did not inhibit aromatase mRNA, but instead it increased it. This may be because of a compensatory increase in Cyp19 mRNA for inhibition of the aromatase enzyme by letrozole; otherwise, testosterone elevation enhances the action of FSHR on Cyp19 expression, which itself stimulates Cyp19 [30, 31].

In this study, letrozole did not significantly reduce VEGF gene expression, and we could not find the mechanisms of reducing OHSS risk by letrozole. Previous studies have shown a positive association between VEGF and follicle development, and VEGF expression may be strictly dependent on follicle diameter [32]. Additionally, we evaluated non-luteinized follicles, and VEGF secretion is increased in luteinized granulosa cells. Since VEGF increases after the luteinizing hormone surge in vivo, further studies are needed to reveal whether hCG administration enhances the effect of letrozole on VEGF suppression.

## Conclusions

Letrozole may promote follicle development by increasing the expression of FSHR. Letrozole may be useful for fertility preservation of patients with estrogen-dependent cancers such as breast cancer and various other cancers. Whether letrozole has a direct effect in reducing OHSS should be explored in further investigations.

## Methods

### Animals and cell culture

Female ICR mice were obtained from Sankyo Labo Service Corporation (Sapporo, Japan). Mice were handled following the guidelines provided by Sapporo Medical University and the Scientists Center for Animal Welfare. Animal protocols were approved by the Sapporo Medical University Institutional Animal Care and Use Committee. Six-week-old female ICR mice (*n* = 4) were euthanized by intraperitoneal injection of pentobarbital (120 mg/kg). Ovaries were removed and secondary follicles (100–160 µm in diameter) were mechanically isolated using 30-gauge needles under an inverted microscope. Follicles with an intact basement membrane, clear granulosa cell layers and oocytes, and centrally located round oocytes were selected. Each follicle was placed individually into wells of a 48-well multiple cell-repellent surface plate (Greiner Bio-One International GmbH, Kremsmünster, Austria) to eliminate factors that regulate follicle growth and steroidogenesis, such as pituitary gonadotropins, steroid hormones, and local growth factors. Each well contained 300 µL of Alpha Minimum Essential Medium (Thermo Fisher Scientific, Waltham, MA, USA) supplemented with 5% fetal bovine serum (Corning, Corning, NY, USA), 6 µg/mL insulin, 5.5 µg/mL transferrin, 6.7 ng/mL sodium selenite, 200 IU/mL penicillin (Thermo Fisher Scientific), and 33 mIU/mL follicle-stimulating hormone (FSH) (Sigma-Aldrich, St. Louis, MO, USA). A previous study showed that the minimal FSH concentration required to elicit a maximal FSH-induced growth response was 67 mIU/mL [33]. Follicles were cultured at 37°C in a humidified environment containing 5% CO_2_. Every other day, half of the culture medium was exchanged with fresh medium. Culture was continued for 10 days.

To evaluate the effects of letrozole on early folliculogenesis, follicles from four mice (12 follicles/mouse/group) were randomly assigned to one of three culture conditions: 1) control (CTRL) group, base media plus letrozole vehicle (dimethyl sulfoxide, DMSO); 2) LET 30 group, CTRL media plus 30 ng/mL letrozole and DMSO; and 3) LET 100 group, CTRL media plus 100 ng/mL letrozole and DMSO. Letrozole was purchased from Tokyo Chemical Industry (Tokyo, Japan). We decided the concentrations of letrozole treatment in this study according to the interview form of letrozole provided by the Novartis Pharma Japan. The form indicates that the serum concentration of letrozole is 10–100 ng/ml in women administered 2.5mg a day for 7 days (almost the same as a schedule for ovarian stimulation).

### Follicle survival and growth

Follicle survival and growth were assessed at days 1, 5, and 10 using an SMZ18 inverted microscope system (Nikon, Tokyo, Japan). Follicles were considered to be degenerating if oocytes became dark or were ejected outside of the follicle, if granulosa cells were dark and lysed, or if the diameter of the follicle decreased. The diameter of each follicle was determined as the average of two perpendicular measurements using NIS Elements Documentation D 3.22.00 (Nikon).

### Measurement of E2 and VEGF

Concentrations of E2 in the culture media were measured at day 10 using an estradiol ELISA test kit (Neogen, Lansing, MI) with a detection range of 0–2.0 ng/mL following the manufacturer’s instructions. VEGF levels in the culture media were measured using a VEGF ELISA test kit (R&D Systems, Abingdon, UK), with a detection range of 0–500 pg/mL, following the manufacturer’s instructions. Interassay and intraassay coefficients of variance in these kits were below 10%.

### RNA extraction, reverse transcription, and real-time quantitative polymerase chain reaction

At day 10 of culture, six to ten follicles in each experimental group were analyzed for mRNA expression. Each follicle was ruptured using a 30-gauge needle, and the follicle wall and cumulus cells were collected for RNA extraction. Total RNA was isolated using the Absolutely RNA Nanoprep Kit (Agilent, Santa Clara, CA, USA) following the manufacturer’s instructions. Complementary DNA was synthesized from 1 µg of total RNA using the Superscript II Reverse Transcriptase kit (Thermo Fisher Scientific). Quantitative polymerase chain reaction was carried out using a TaqMan gene expression assay and AB StepOne Plus Real-Time PCR System (Thermo Fisher Scientific). Gene expression levels of FSH receptor (*Fshr*) (Assay ID: Mm00442819_m1), aromatase (cytochrome P450, family 19, subfamily a, polypeptide 1, *Cyp19a1*) (Assay ID: Mm00484049_m1), and vascular endothelial growth factor A (*Vegfa*) (Assay ID: Mm00437306_m1), and 18S ribosomal RNA (Assay ID: Mm03928990_g1), as the normalization control were analyzed. The amplification program included 40 cycles of denaturation at 95°C for 15 s and 60°C for 60 s. All reactions were run in triplicate. Gene expressions were calculated by the 2^−ΔΔCt^ method.

### Data analysis

Data are presented as mean ± standard error of the mean (SEM). Statistical significance was determined using one-way analysis of variance (ANOVA) and Student–Newman–Keuls post-hoc analysis with SigmaPlot version 13.0 (Systat Software, San Jose, CA, USA) for comparison of data among different treatment groups. *P* < 0.05 indicated statistical significance.

## Data Availability

We are not sharing the data and material used in this manuscript, as they will be used for subsequent research.

## Abbreviations

DMSO: dimethyl sulfoxide
E2: estradiol
VEGF: vascular endothelial growth factor
FSHR: follicle-stimulating hormone receptor
*Cyp19a1*: cytochrome, P450, subfamily a, polypeptide 1
PCOS: polycystic ovary syndrome
COS: controlled ovarian stimulation
OHSS: ovarian hyperstimulation syndrome
hCG: human chorionic gonadotropin
